# Bridging the basic biomedical researches and clinical practices with biomedical informatics

**DOI:** 10.1186/1479-5876-10-S2-A52

**Published:** 2012-10-17

**Authors:** Lei Liu, Zuofeng Li

**Affiliations:** 1 Shanghai Center for Bioinformation Technology, Shanghai, China; 2 Shanghai Academy of Science and Technology, Shanghai, China

## 

The emergence of genome-wise high-throughput technologies have led to the creation of the interdisciplinary field between biotechnology and information technology, where bioinformatics came out. With the application of these technologies in medical research, the concept of "translational medicine" has attracted more and more attentions and concerns. How can we translate the results of basic biomedical research into clinical practices? How should we promote basic research to meet the demands of modern medicine? These questions are the core issues in translational medicine. To solve these problems, information technology is the key. The integration and interaction among biotechnology, medicine and information technology leads to the formation of Biomedical Informatics.

The challenges of building modern medical information systems are to reduce the redundancy of the data and functions, to seek the Inter-connection and Inter-communication technology for different data structures and system architectures in the complex business environment, and to build an efficient and flexible content management system. To meet these challenges, we need to build a high-quality information system integrating clinical data and basic biomedical research data, which is the core of translational medicine and the key to obtain the transformation “from the laboratory bench to hospital bed”. This information system should include three parts: de-identified clinical data repository (de-id CDR), “omics” databases including analysis platforms and tissue banks (diseases and normal controls) adhering to the requirements of research design.

The health information technologies related to clinical data acquisition, storage, management, and applications are essential for the 21st century medical and health services. The automatic or semi-automatic form filling for research by importing and integrating data from different information systems, e.g. PACS, HIS, EMR and LIS, can significantly improve the data accuracy and work efficiency. However, in current medical information systems, a large number of important clinical data exist only in free text documents and reports. To extract these data, medical natural language processing technology has become a hot and challenging field in biomedical informatics research. The automatic processing of medical text in English has been studied for many years, and many tools have been developed. But in China, research on automatic processing of Chinese medical documents has rarely been reported. Considering the sheer size of the population in China, Chinese medical language processing technology may be an important subject in translational medicine research in China.

Translational medicine research depends on a large cohort of human tissue samples. Tissue bank is the core of clinical research information platform. The efficiency of procedures about disease prediction, personalized treatment, and assessment studies will be judged on the protein or gene level using human tissue, blood and body fluids. The research results of these biological macromolecules in human tissues will directly determine their values in clinical practices and commercial prospects. The establishment of high-quality and high-level information systems is one of the foundations of translational medicine.

